# Supplementary figures and images for: Cell–substrate adhesion drives Scar/WAVE activation and phosphorylation by a Ste20-family kinase, which controls pseudopod lifetime
Source: PLoS Biol. 2020 Aug 3;18(8):e3000774. doi: 10.1371/journal.pbio.3000774 (PMC7425996; doi:10.1371/journal.pbio.3000774)

**A**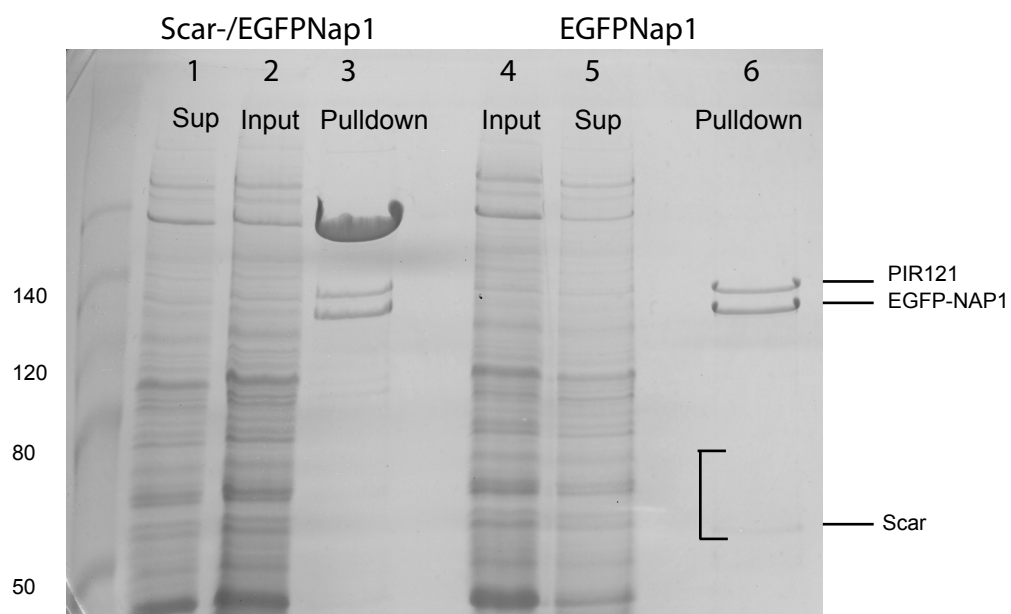**B**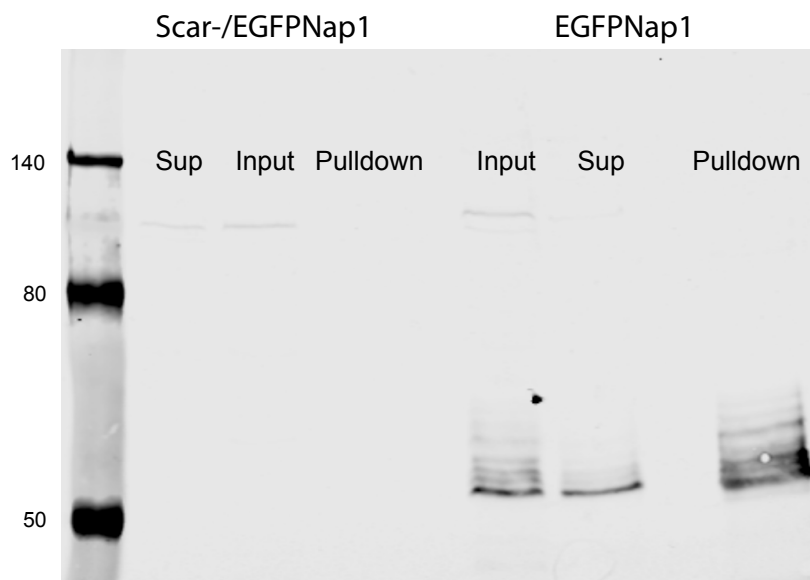**C**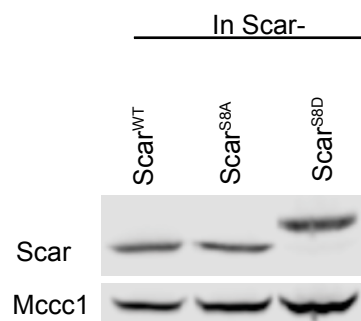

Supplement: S1 Fig — (A) Coomassie brilliant blue stained low-bis acrylamide PAGE gel of GFP-TRAP pull-down samples. Lane 1, 2, and 3 indicates samples from Scar-/EGFPNAP1, and lane 4, 5, and 6 indicates samples from EGFPNap1 cells. The Scar band indicated on the gel was excised for LC-MS/MS. (B) Representative western blot of above gel indicating phosphorylated Scar bands. LC-MS/MS, liquid chromatography-tandem mass spectrometry. (PDF) [file pbio.3000774.s001.pdf]

**A**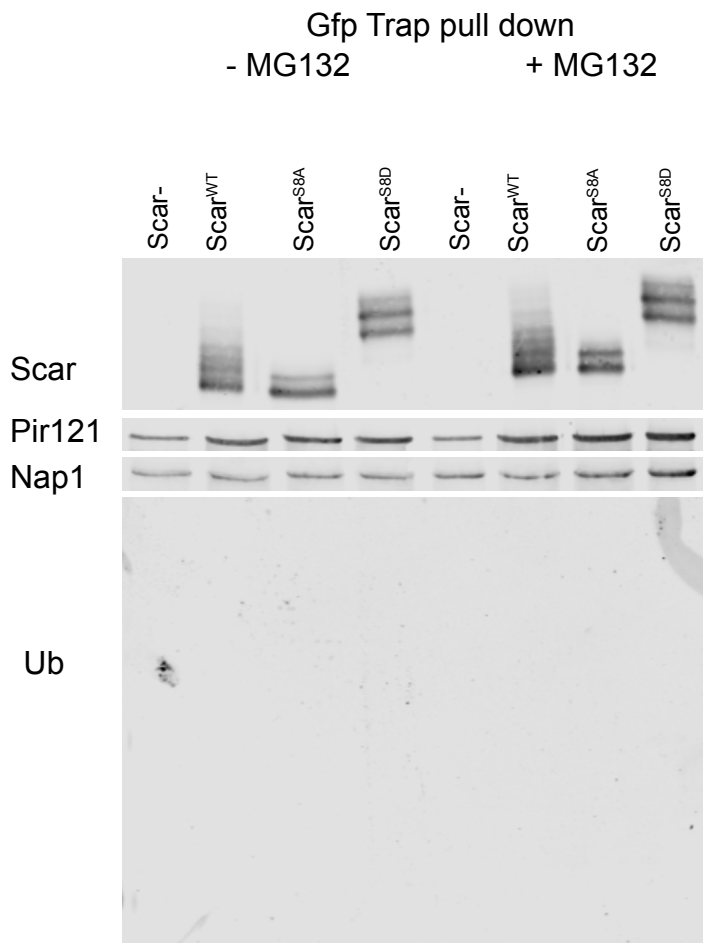**B**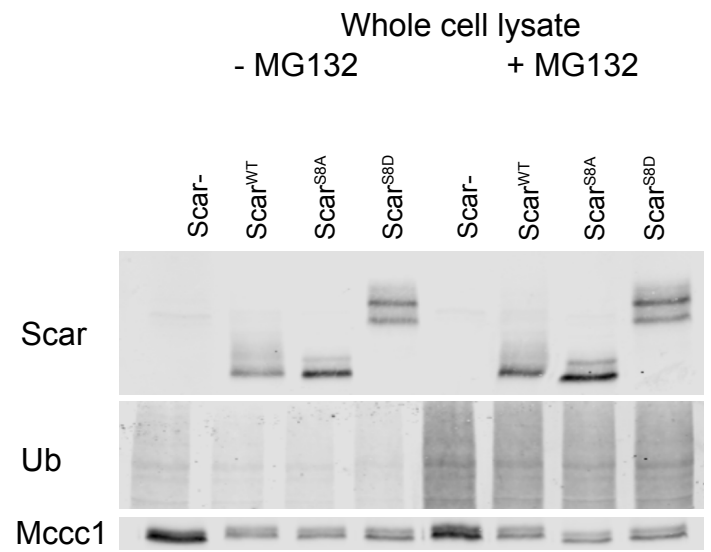

Supplement: S2 Fig — (A) GFP-TRAP pull-down was performed using MG132-treated and untreated Dictyostelium Scar-/EGFP-NAP1 cells expressing ScarWT, ScarS8A, and ScarS8D. The eluates were analyzed on low-bis PAGE western blotting for Pir121/Nap1/Scar and ubiquitin. (B) Scar expression in the cell lysates was analyzed by low-bis PAGE and western blotting. (PDF) [file pbio.3000774.s002.pdf]

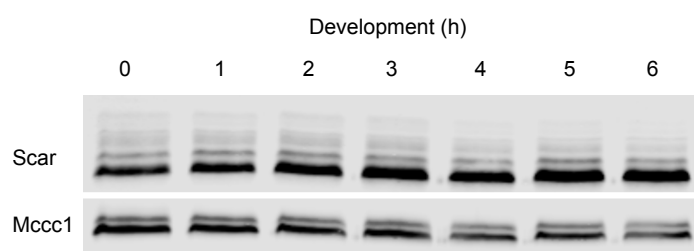

Supplement: S3 Fig — Cells were washed with non-nutrient buffer, starved for the indicated times, separated on low-bis gels, and probed with anti-Scar. The amount of phosphorylated Scar is reduced following development. (PDF) [file pbio.3000774.s003.pdf]

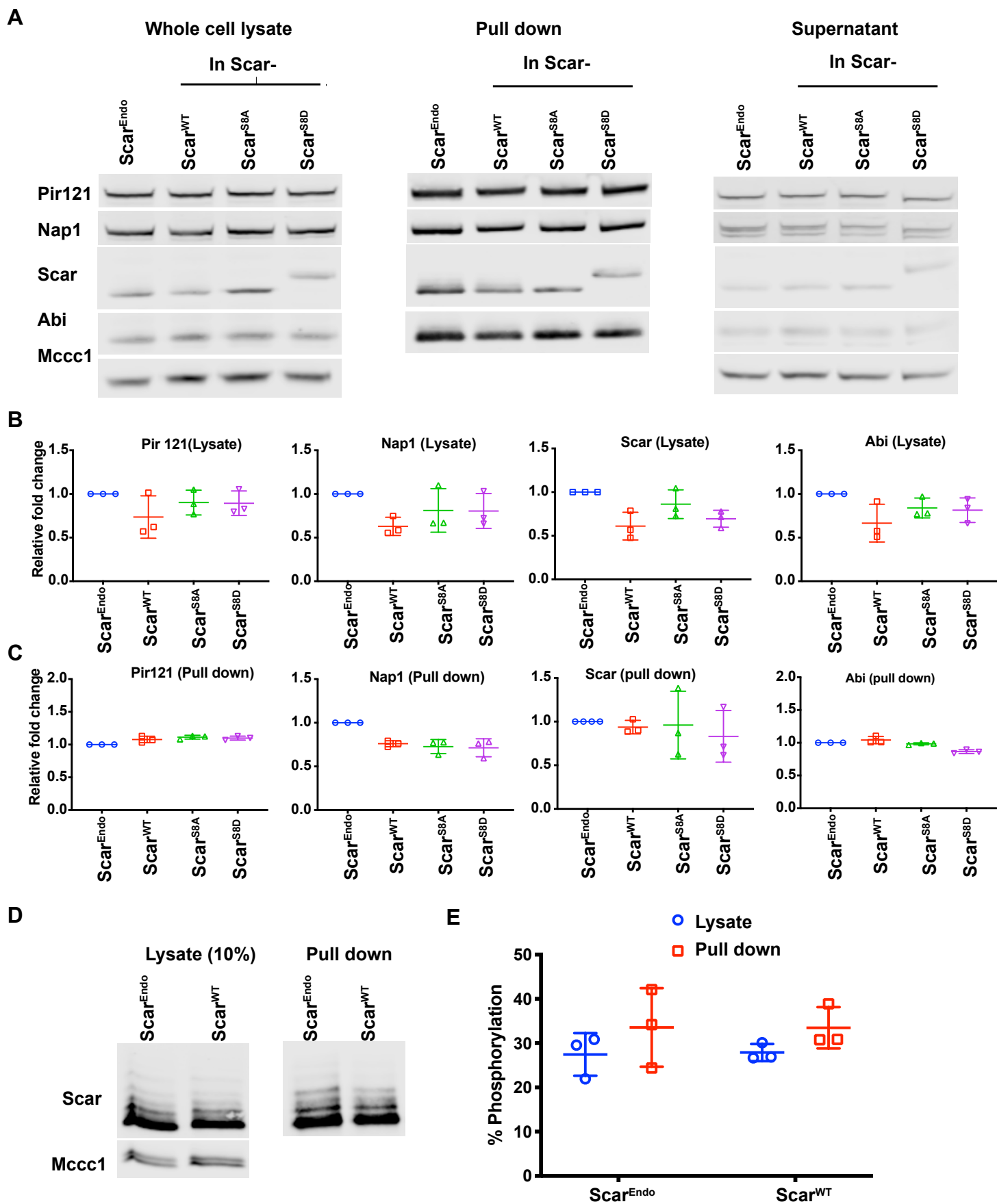

Supplement: S4 Fig — Nap-/EGFP-Nap1 and Scar-/Nap-/EGFP-Nap1 cells rescued with ScarWT, ScarS8A, and Scar8D cell lysates were immunoprecipitated using GFP-TRAP. (A–C) Lysate and pull-down samples were analyzed for the expression of Pir121, Nap1, Scar, and Abi. Quantification of western blots shows that similar to ScarEndo, ScarWT, ScarS8A, and Scar8D formed stable complexes. The numerical data are included in S1_Data. (D–E) Phosphorylated Scar in the complex. Lysate and GFP-TRAP samples were analyzed on low-bis gels. ScarEndo and ScarWT are similarly phosphorylated in lysates and GFP-TRAP samples. The numerical data are included in S1 Data. (PDF) [file pbio.3000774.s004.pdf]
