# Supplementary material for: Cell–substrate adhesion drives Scar/WAVE activation and phosphorylation by a Ste20-family kinase, which controls pseudopod lifetime
Source: PLoS Biol. 2020 Aug 3;18(8):e3000774. doi: 10.1371/journal.pbio.3000774 (PMC7425996; doi:10.1371/journal.pbio.3000774)

Fig 1

Singh et al

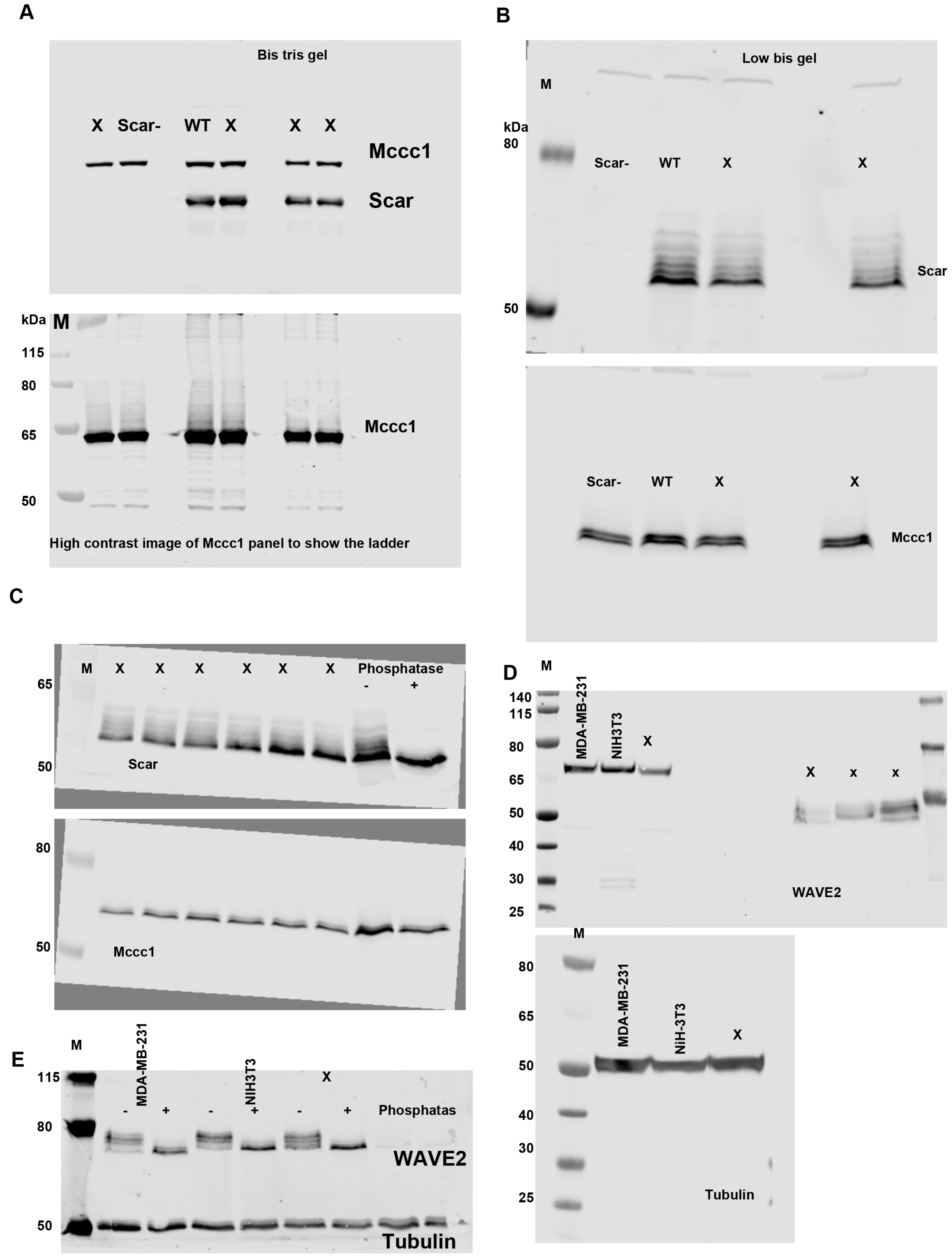

Blots in all figures are scanned with Li-Cor imaging system.

Fig 1 Singh et al

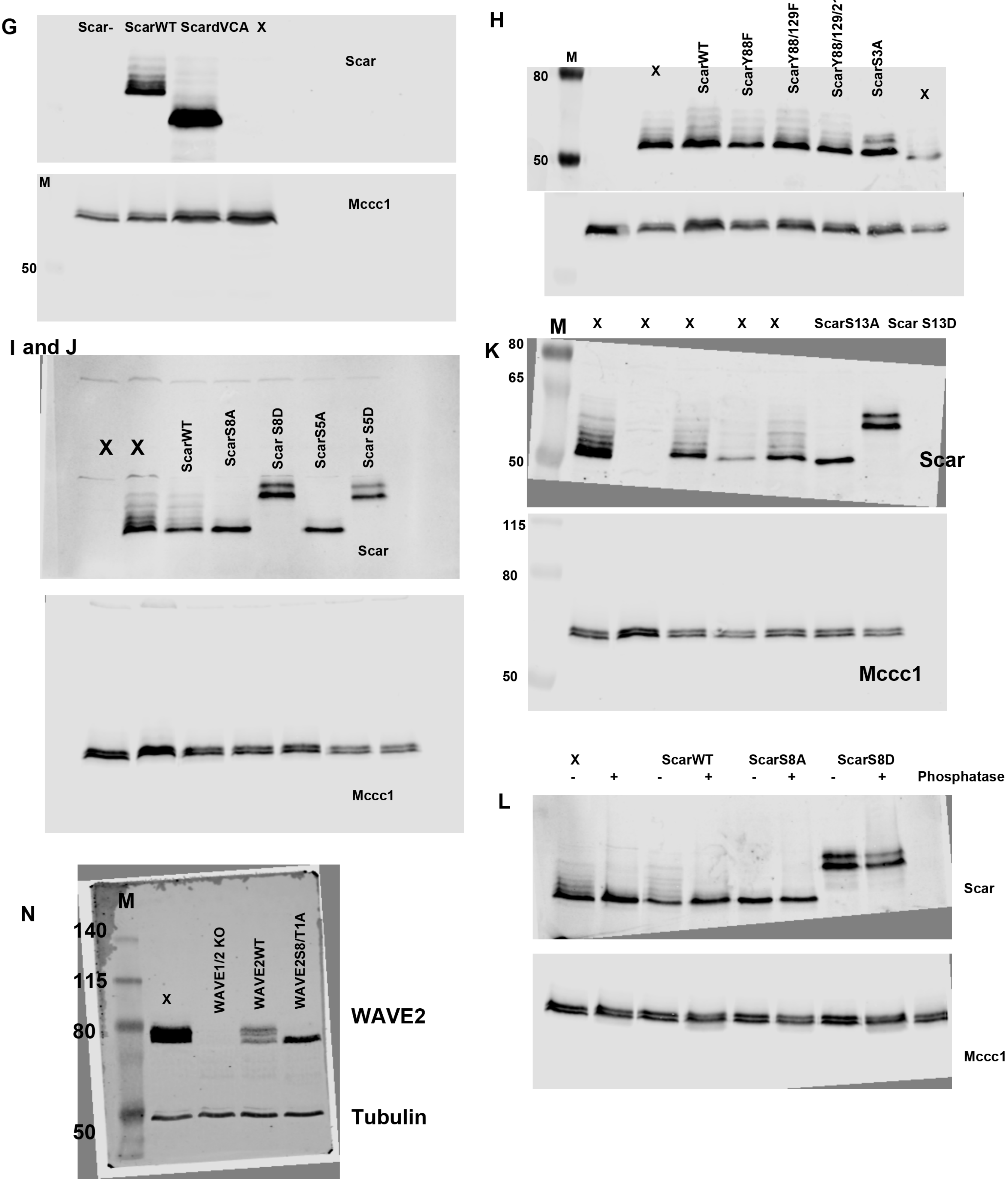

Fig 2

Singh et al

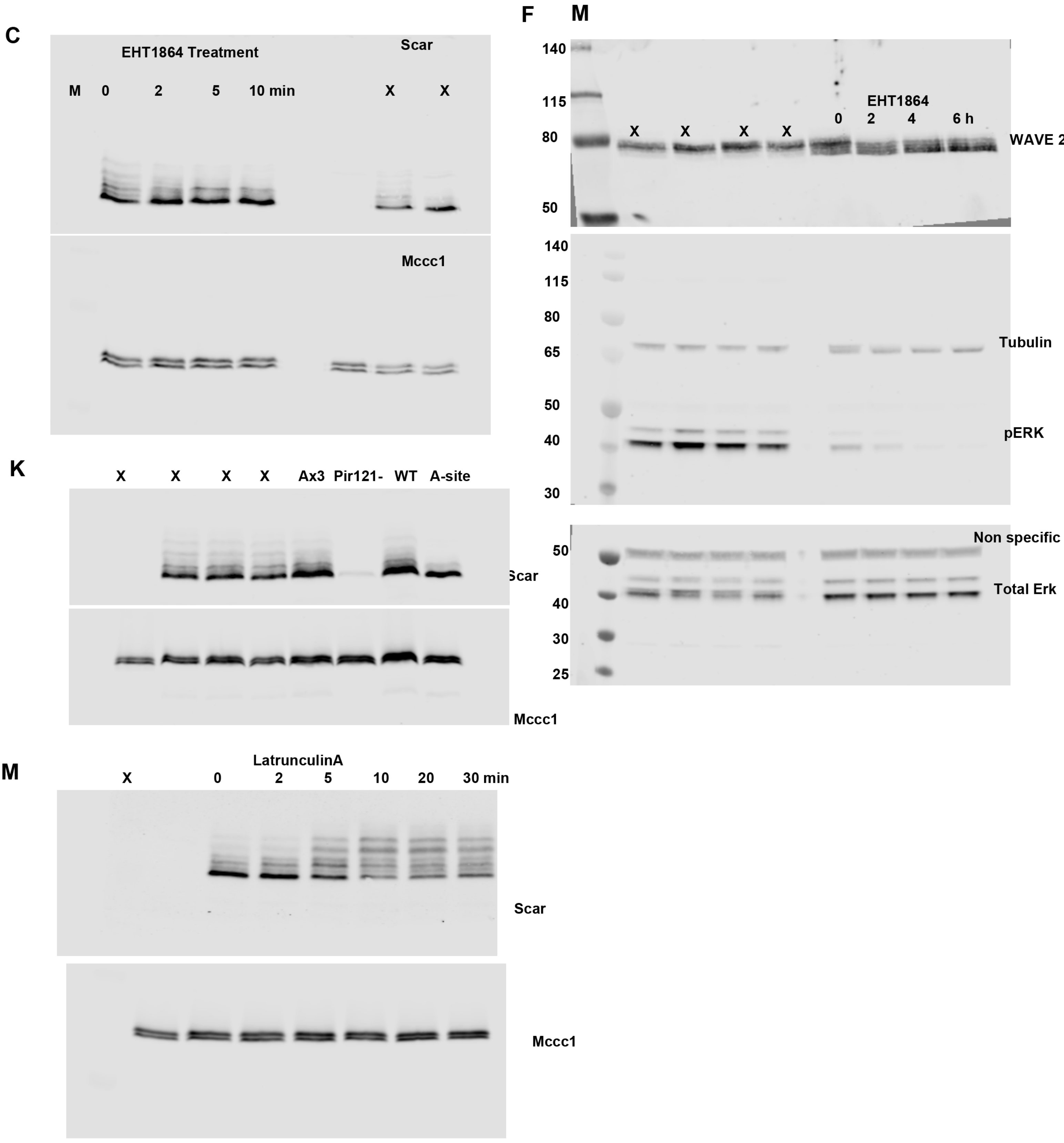

Blots were scanned with Li-cor

Fig 3

Singh et al

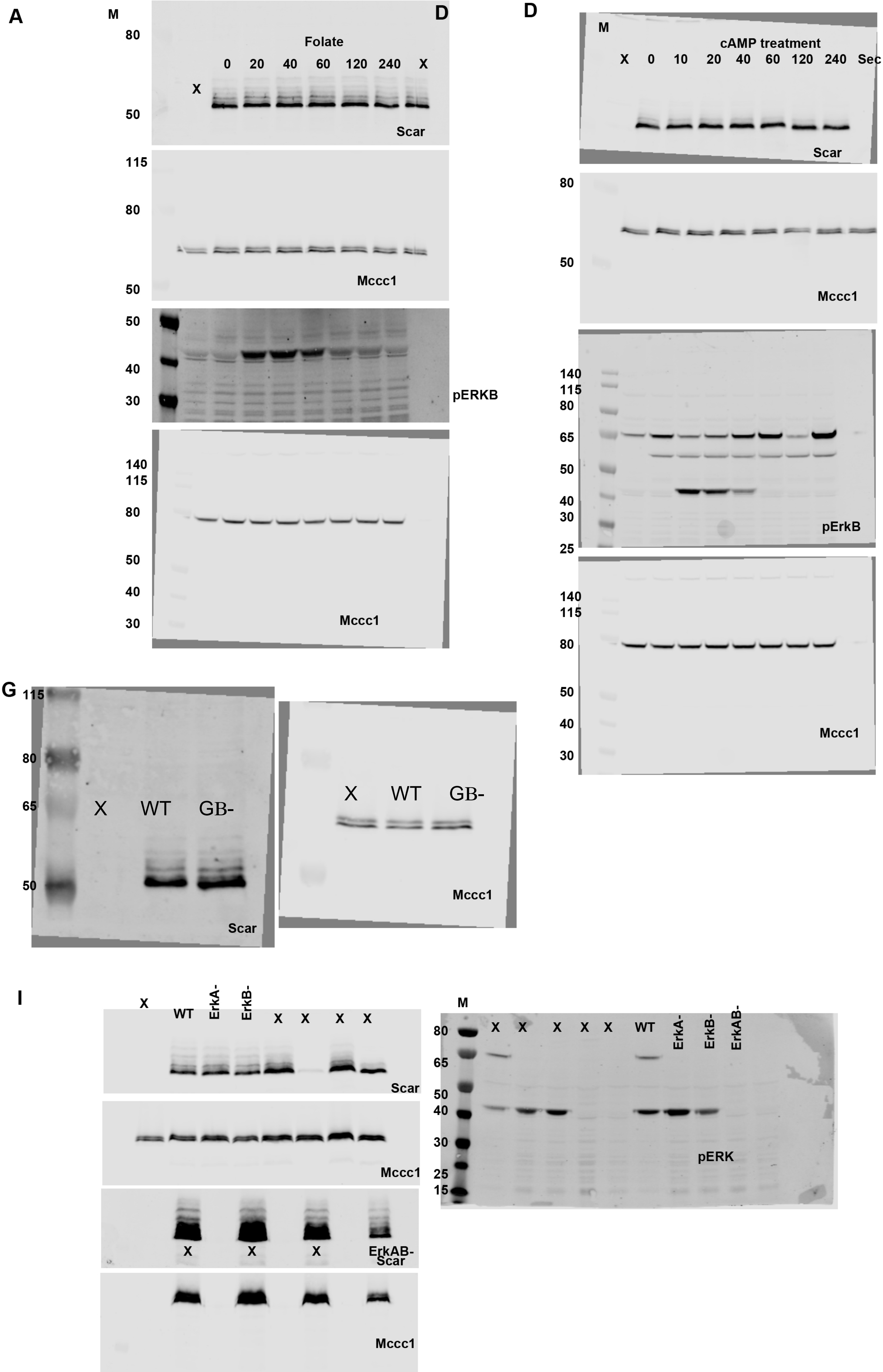

Fig 3

Singh et al

K

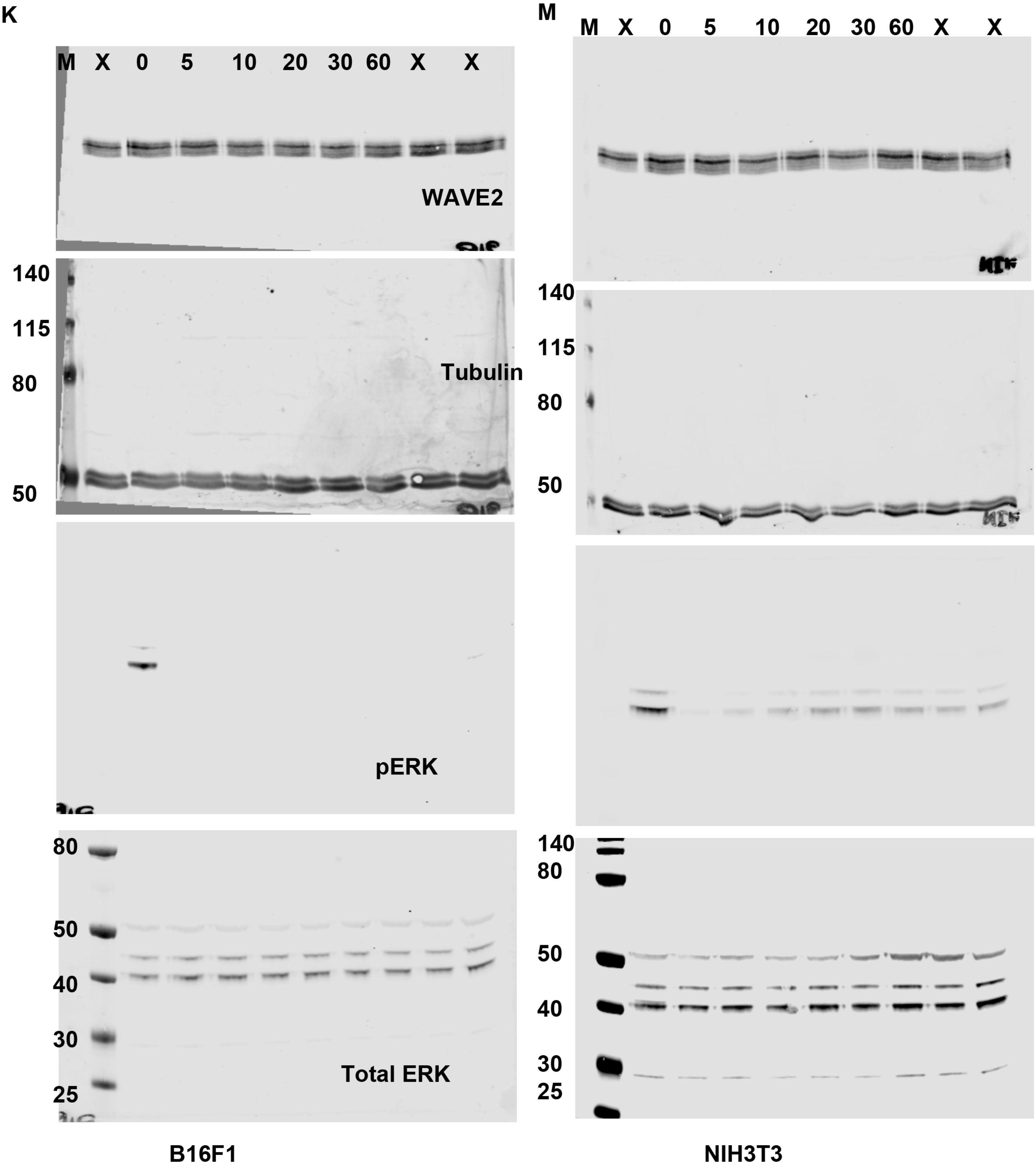

Fig 4 Singh et al

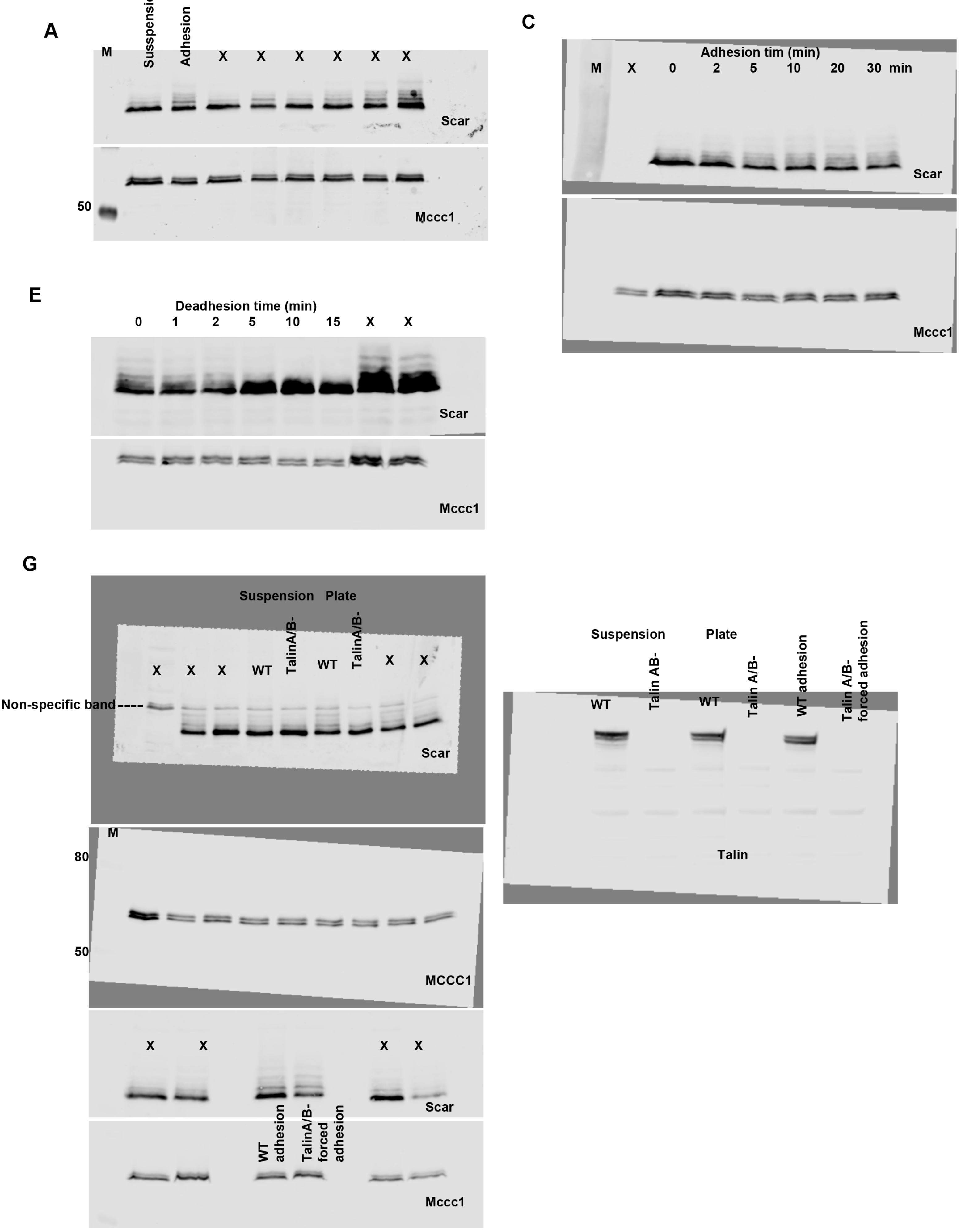

Fig 7

A

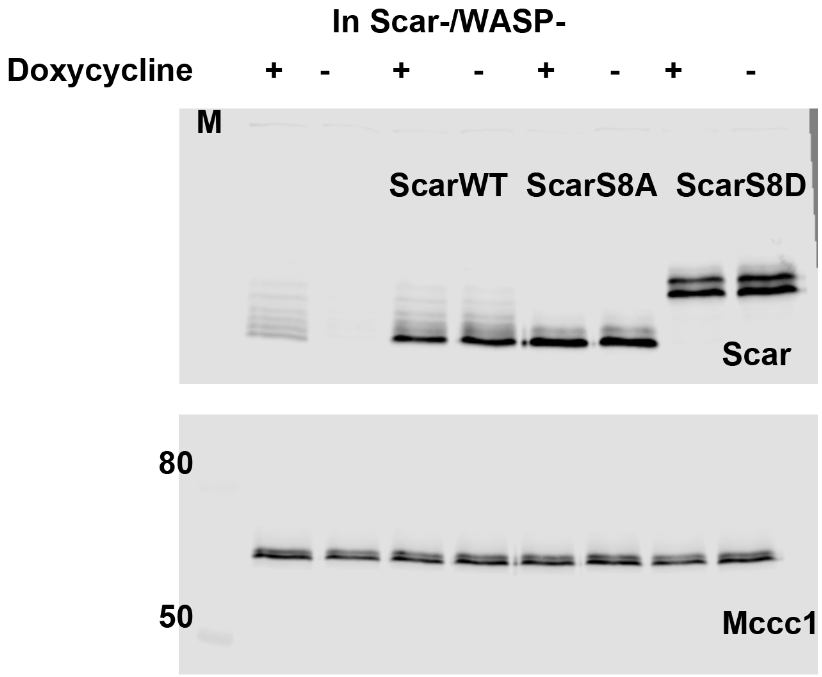
In Scar-/WASP-
Doxycycline + - + - + - + -
M
ScarWT
ScarS8A
ScarS8D
Scar
80
50
Mccc1

Fig 8

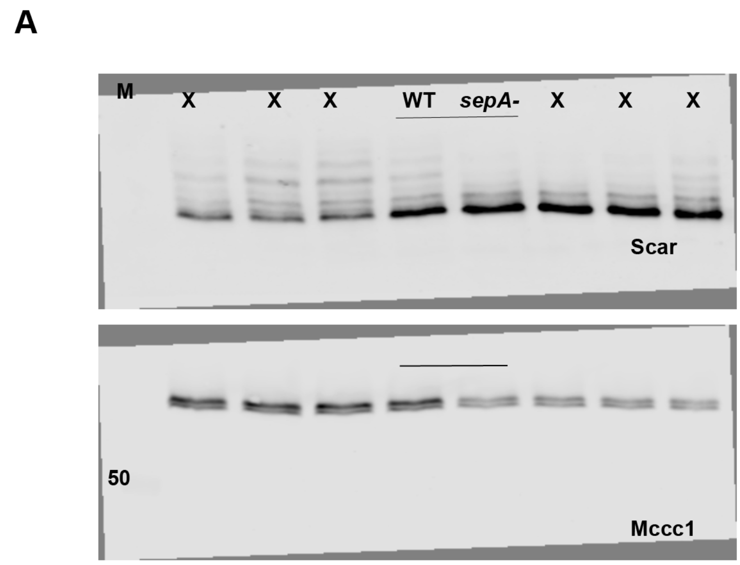
A
M
X
X
X
WT
sepA-
X
X
X
Scar
50
Mccc1

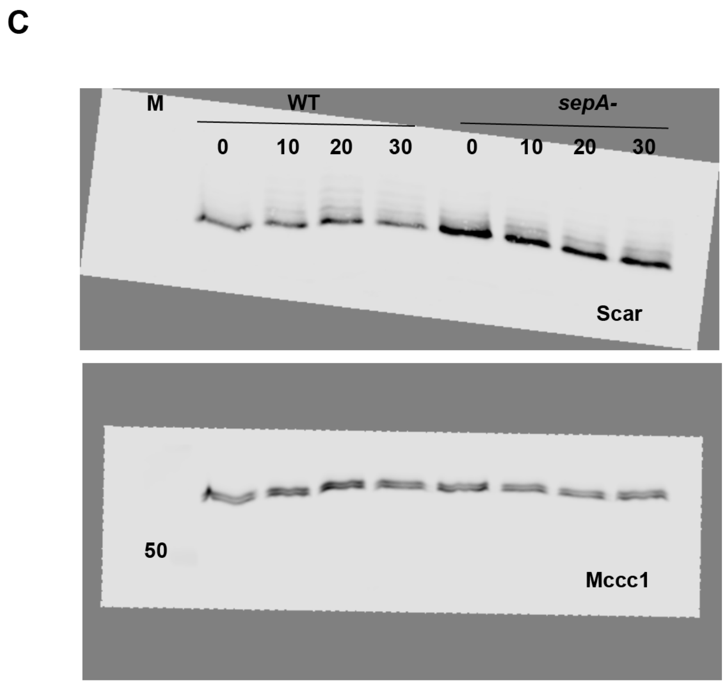
C
M
WT
sepA-
0
10
20
30
0
10
20
30
Scar
50
Mccc1

S1 Fig.

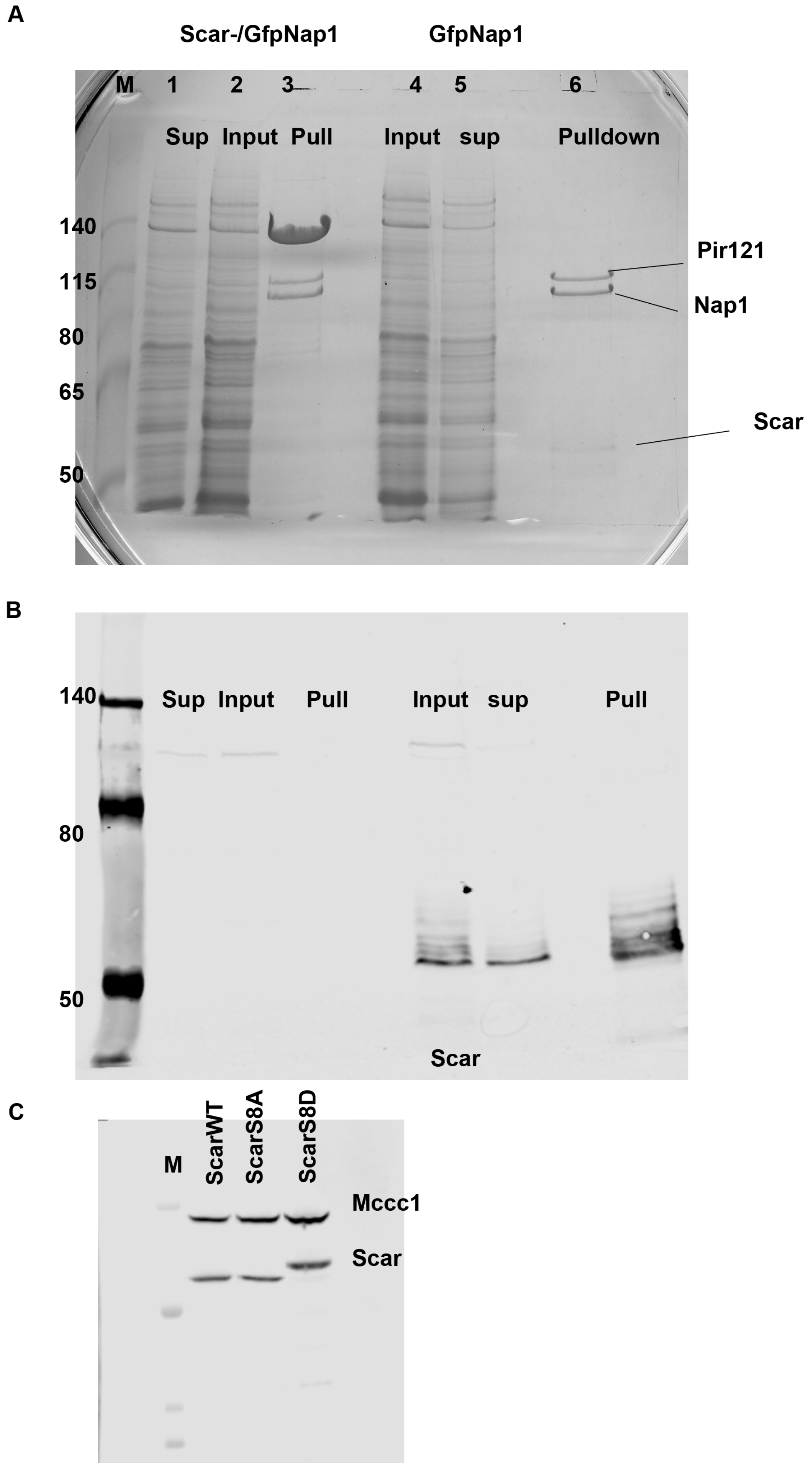
A
Scar-/GfpNap1
GfpNap1
M
1
2
3
4
5
6
Sup
Input
Pull
Input
sup
Pulldown
140
115
80
65
50
Pir121
Nap1
Scar
B
140
80
50
Sup
Input
Pull
Input
sup
Pull
Scar
C
M
ScarWT
ScarS8A
ScarS8D
Mccc1
Scar

S2 Fig

Singh et al

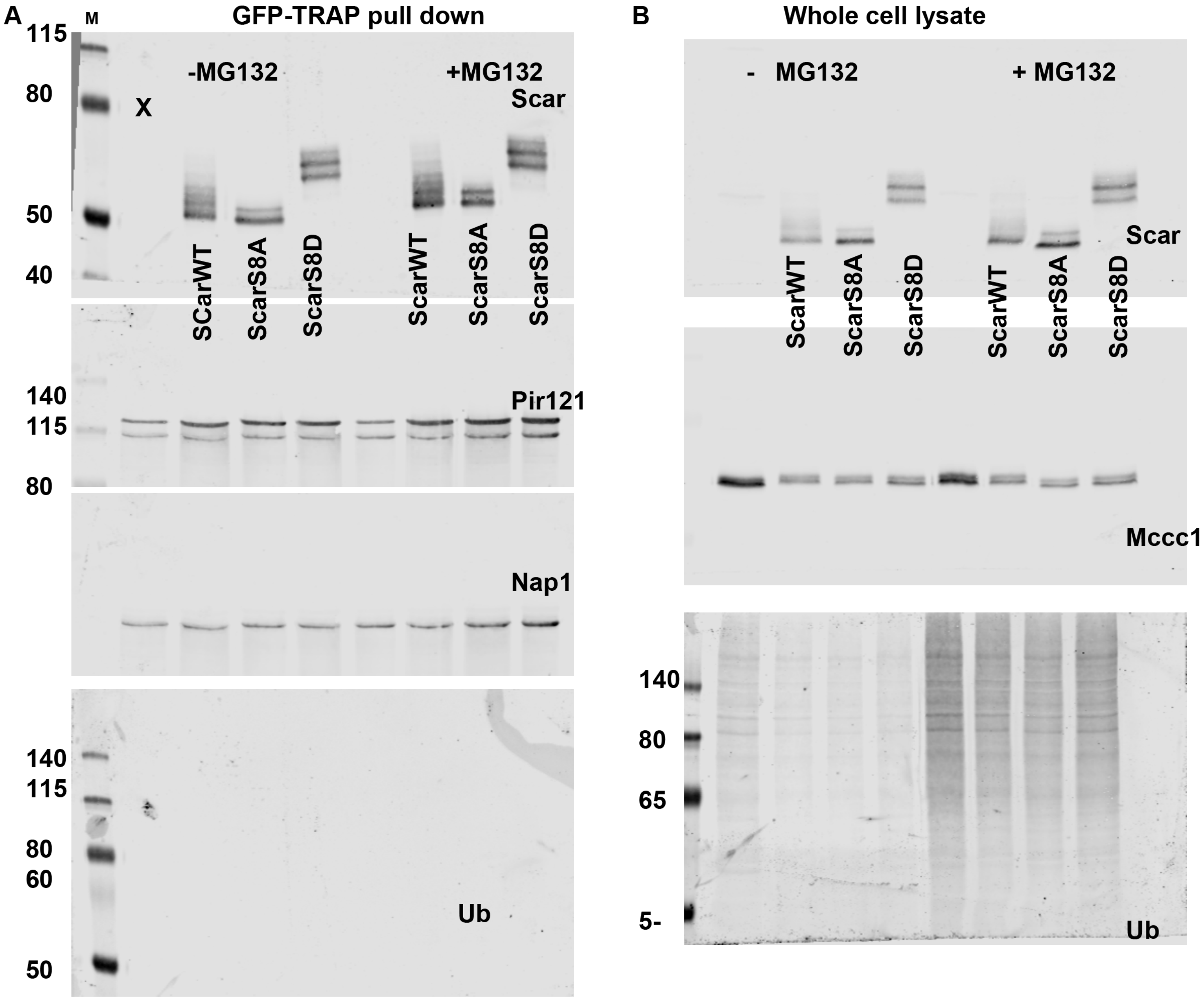

S3 Fig

Singh et al

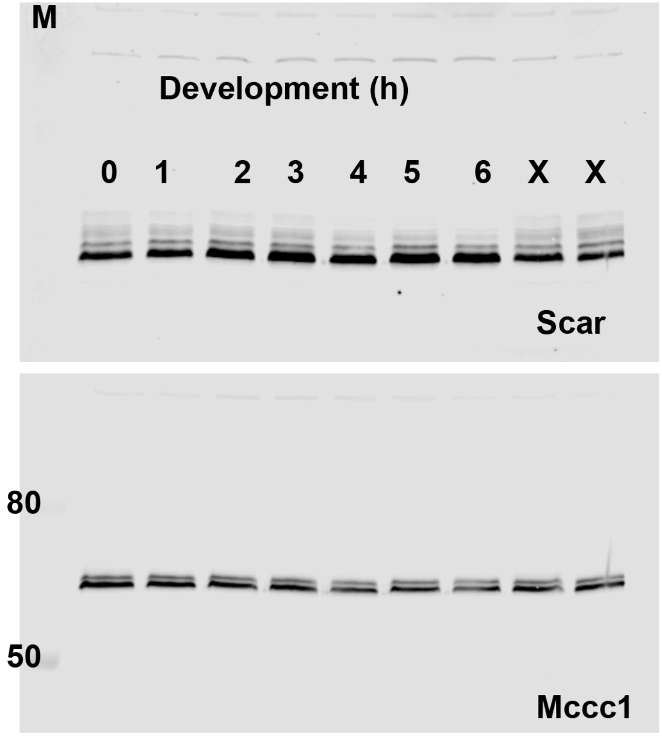

S4 Fig

Singh et al

A

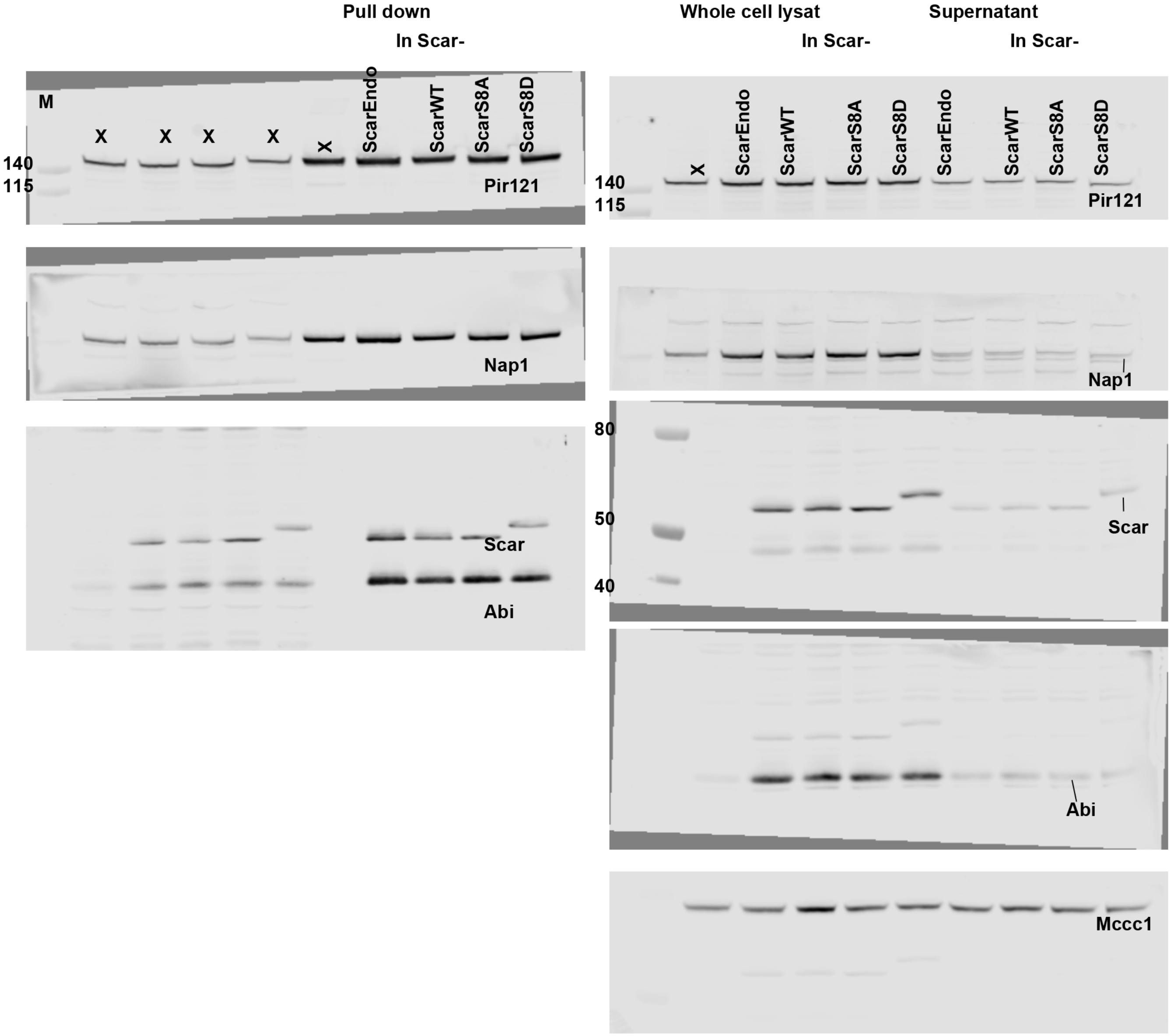

D

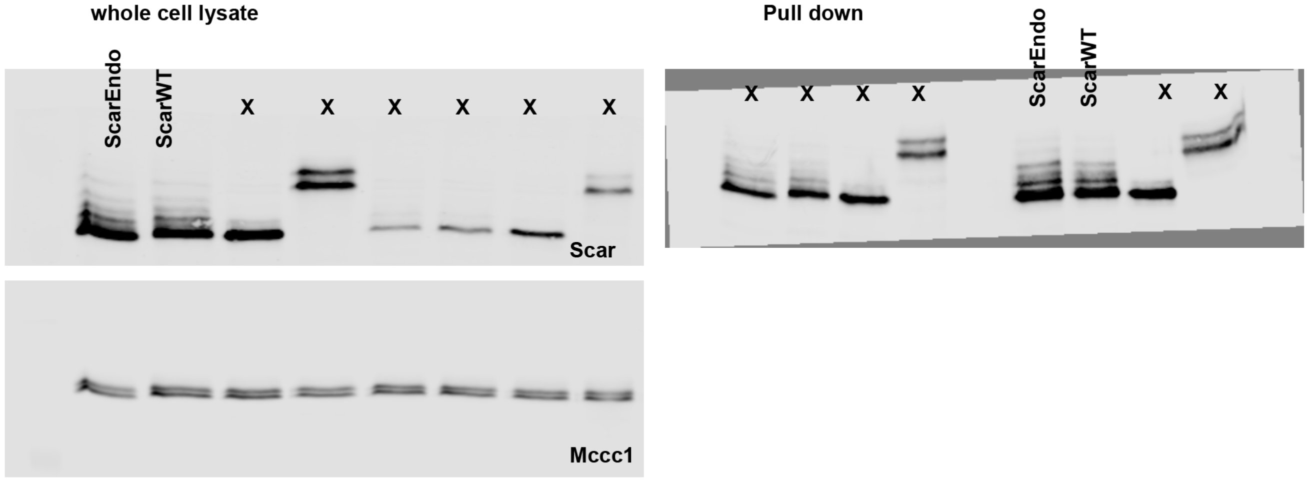

Supplement: S1 Raw images — (PDF) [file pbio.3000774.s016.pdf]
