# Supplementary material for: Cell–substrate adhesion drives Scar/WAVE activation and phosphorylation by a Ste20-family kinase, which controls pseudopod lifetime
Source: PLoS Biol. 2020 Aug 3;18(8):e3000774. doi: 10.1371/journal.pbio.3000774 (PMC7425996; doi:10.1371/journal.pbio.3000774)
Supplement: S1 Table. Peptide sequences and highlighted phosphorylated residues — (DOCX) [file pbio.3000774.s018.docx]

**S1 Table**: Peptide sequences and highlighted phosphorylated residues.

| **Phosphorylated Residues in Scar** | **Peptide Sequence** | **Identification Source** |
| --- | --- | --- |
| Y88 | PSIEDYHRNTS | By MS and Screening |
| Y129 | SINTVYEKCKP | By MS and Screening |
| Y210 | VTKVRYDPVTG | By MS and Screening |
| S287/S290 | PPLNTSTPSPSSSF | By MS and Screening |
| S301 | QGRPPSTGFNT | By MS and Screening |
| S335/S339 | ANNRLSVHNSAPIVA | Screening |
| S384/S388/S389 | ASGARSDLLSSIMQGM | By MS and screening |
| **Phosphorylated Residues in WAVE2** | | |
| S293/S296/S298 | GPKRSSVVSPSHPPPA | Chen et al., 2010, Lebensohn and Kirschner 2009, Phosphosite.org, |
| S308 | APPLGSPPGPK | Lebensohn and Kirschner 2009 |
| S343/T346/S351 | PVGFGSPGTPPPPSPPSFP | Mendoza et al., 2011, Danson et al., 2007 |
| S429 | TKPKSSLPAVS | Mertins et al., 2016, Phosphosite.org. |
| S442 | RSDLLSAIRQG | Mertins et al., 2016, Phosphosite.org. |
