# Supplementary material for: Cell–substrate adhesion drives Scar/WAVE activation and phosphorylation by a Ste20-family kinase, which controls pseudopod lifetime
Source: PLoS Biol. 2020 Aug 3;18(8):e3000774. doi: 10.1371/journal.pbio.3000774 (PMC7425996; doi:10.1371/journal.pbio.3000774)
Supplement: S2 Table — (DOCX) [file pbio.3000774.s019.docx]

# S2 Table: List of reagents, cells and plasmids used in this study.

| Reagent or Resource | Source | | Identifier |
| --- | --- | --- | --- |
| Antibodies | | | |
| Scar | [1] | | NA |
| WAVE2 | Cell signalling | | RRID:AB_2216981 |
| Alpha-Tubulin | Sigma | | T6199 |
| Erk | Cell signalling | | 9107s |
| pErk | Cell signalling | | 9101s |
| Ubiquitin (Pan) | MERK | | MABS486 |
| TalinB | This lab | | NA |
| Goat anti rabbit IgG (H+L) Dylight 800 | Invitrogen | | SA535571 |
| Rabbit anti Sheep IgG (H+L) Dylight 800 | Invitrogen | | SA510060 |
| Streptavidin Alexa 680 conjugate | Thermo Fisher Scientific | | S32358 |
| Chemicals, Peptides, and Recombinant Proteins | | | |
| Alexa-568 phalloidin | Thermo Fisher Scientific | | A12380 |
| Laminin | Sigma-Aldrich | | L2020 |
| Lipofectamine-2000 | Thermo Fisher Scientific | | 116680-19 |
| Paraformaldehyde | Sigma-Aldrich | | P6148 |
| DAPI Fluoromount-G | SouthernBiotech | | 0100-20 |
| Latrunculin A | Sigma-Aldrich | | L5163 |
| EHT1864 (Rac1 inhibitor) | Sigma-Aldrich | | E1657 |
| Uo126 | Sigma-Aldrich | | 662005 |
| MG132 | Sigma-Aldrich | | M8699 |
| Lambda Phosphatase | NEB | | P0753S |
| Protein estimation assay | | | |
| Precission red Protein assay reagent | Cytoskeleton | | ADV02 |
| Experimental models: Organisms |  | | |
| Dictyostelium discoideum cells | | | |
| Wild type strain | Dicty stock center | | Ax3 |
| Wild type strain | Gift from Robert R. Kay | | Ax2 |
| Talin A-/B- | Dicty stock center | | DBS0304581 |
| ErkA- | Nichols and Paschke et al., 2019 | | HM1733 |
| ErkB- | Nichols and Paschke et al., 2019 | | HM1734 |
| ErkA-/B- | Nichols and Paschke et al., 2019 | | HM1940 |
| Scar- | Blagg et al., 2003 | | Ir46; DBS0236926 |
| Scar-/EGFP-Nap1 | Ura et al., 2012 | | NA |
| EGFP-Nap1 | Ura et al., 2012 | | NA |
| Pir121-/Pir121-EGFP | Sachks et al., 2018 | | SP7 |
| Pir121-/Pir121-K193D/R194D | Sachks et al., 2018 | | SP11 |
| Gβ- | This Lab | | JK30 |
| sepA- | Dicty stock centre | | DBS0350906 |
| Mammalian cell lines | | | |
| B16F1 | Gift from Prof. Klemens Rottner | | NA |
| B16-F1 WAVE1/2 KO clone #11/20 | This study | | MSw11/20 |
| Recombinant DNA | | | |
| Modular expression vector | | [2] | pDM304 |
| Modular expression vector- pCDNA3.1 | | ThermoFisher Scientific, Gift from Prof. Laura Machesky | V79020 |
| pSpCas9(BB)-2A-Puro | | Addgene | 48139 |
| Scar WT expression | | This Study | pSP127, pSP210 |
| ScarY88F expression | | This Study | pSP143 |
| Scar Y88,129F expression | | This Study | pSP144 |
| Scar Y88,129, 210 F Expression | | This Study | pSp145 |
| Scar^S3A^ expression vector | | This Study | pSP146 |
| ScarS5A and lifeact-mRFPmars2 co-expression vector | | This Study | pSP199 |
| ScarS5D lifeact-mRFPmars2 expression | | This Study | pSP206 |
| Scar^S8A^ expression | | This Study | pSP263 |
| Scar S8D expression | | This study | pSP264 |
| WT Scar and Life-act-mRFPMars2 co-expression | | This study | pSP151, pSP210 |
| Scar S8A and Life-act-mRFPMars2 co-expression | | This Study | pSP203, pSP212 |
| Scar S8D and Life-act-mRFPMars2 co-expression | | This Study | pSP208, pSP214 |
| Scar S13A and Life-act-mRFPMars2 co-expression | | This Study | pSP257 |
| Scar S13D and Life-act-mRFPMars2 co-expression | | This Study | pSP258 |
| Scar WT and HSPC300-EGFP co-expression vector | | This Study | pSP229 |
| Scar S8A and HSPC300-EGFP co-expression vector | | This Study | pSP231 |
| ScarS8D and HSPC300-EGFP | | This Study | pSP233 |
| HSPC300-EGFP | | [3] |  |
| Scar^dVCA^ expression | | [3] | pDM304-Scar^dVCA^ |
| ArpC2-mRFPmars2 expression | | This study | pSP260 |
| HSPC300-EGFP expression | | This study | pSP261 |
| Pak-CRIB-mRFPmars2 expression vector | | This Study | pSP148 |
| Human WAVE2 expression vector | | This Study | pSP330 |
| Human WAVE2^S8A/T1A^ | | This Study | pSp350 |
| Software and Algorithms | | | |
| Prism 7 (Graphpad Software, La Jolla, USA) | | | |
| ImageJ FIJJI (NIH) | | | |

1. Blagg SL, Stewart M, Sambles C, Insall RH. PIR121 regulates pseudopod dynamics and SCAR activity in Dictyostelium. Curr Biol. 2003;13(17):1480-7. PubMed PMID: 12956949.

2. Veltman DM, Akar G, Bosgraaf L, Van Haastert PJ. A new set of small, extrachromosomal expression vectors for Dictyostelium discoideum. Plasmid. 2009;61(2):110-8. doi: 10.1016/j.plasmid.2008.11.003. PubMed PMID: 19063918.

3. Ura S, Pollitt AY, Veltman DM, Morrice NA, Machesky LM, Insall RH. Pseudopod growth and evolution during cell movement is controlled through SCAR/WAVE dephosphorylation. Curr Biol. 2012;22(7):553-61. doi: 10.1016/j.cub.2012.02.020. PubMed PMID: 22386315; PubMed Central PMCID: PMCPMC4961229.
