# Supplementary material for: Cell–substrate adhesion drives Scar/WAVE activation and phosphorylation by a Ste20-family kinase, which controls pseudopod lifetime
Source: PLoS Biol. 2020 Aug 3;18(8):e3000774. doi: 10.1371/journal.pbio.3000774 (PMC7425996; doi:10.1371/journal.pbio.3000774)
Supplement: S3 Table — (DOCX) [file pbio.3000774.s020.docx]

# S3 Table: List of oligos used in Scar/WAVE cloning and mutagenesis of unphospho- Scar and phospho-mimetic Scar .

| Oligonucleotides used in Scar cloning and Site directed mutagenesis | | | | |
| --- | --- | --- | --- | --- |
| Name of Primer | | Forward primer | | Reverse Primer |
| Scar | | GGATCCAAAATGGTATTAATTACAAGATATTTACC | | ACTAGTTTAATCCCAATCAGAATCATCAGATTC |
| Y88F | | CAATACCATCGATTGAAGATTTTCATAGAAATACATCAATCGAT | | ATCGATTGATGTATTTCTATGAAAATCTTCAATCGATGGTATTG |
| Y129F | | CGGCATCAATCAATACTGTCTTTGAAAAATGTAAACCACCACC | | GGTGGTGGTTTACATTTTTCAAAGACAGTATTGATTGATGCCG |
| Y210F | | AATCCGTTACAAAGGTACGTTTTGATCCTGTAACTGGTGAGA | | TCTCACCAGTTACAGGATCAAAACGTACCTTTGTAACGGATT |
| S287,290, 301A | | CCACCACCATTAAATACAGCAACCCCTGCACCATCCTCTTCATTCCAAGGTAGACCACCTGCAACTGGTTTCAATACTCC | | GGAGTATTGAAACCAGTTGCAGGTGGTCTACCTTGGAATGAAGAGGATGGTGCAGGGGTTGCTGTATTTAATGGTGGTGG |
| S335,339A | | GAGCTGCAAACAATCGTTTAGCAGTCCATAACGCAGCTCCAATTGTTGCTGCTCCAG | | CTGGAGCAGCAACAATTGGAGCTGCGTTATGGACTGCTAAACGATTGTTTGCAGCTC 3 |
| S384, 388, 389A | | CCAAAAGCATCCGGTGCTCGTGCAGATCTTCTCGCAGCAATTATGCAAGGTATGGCA | | TGCCATACCTTGCATAATTGCTGCGAGAAGATCTGCACGAGCACCGGATGCTTTTGG |
| VCA domain S-A | | GTATTGCTTGGGCTGGTGATGCTGATGCAGCAGAAGATGAAGCAGATGATGCAGATTGGGATTAAACTAGT | | ACTAGTTTAATCCCAATCTGCATCATCTGCTTCATCTTCTGCTGCATCAGCATCACCAGCCCAAGCAATAC |
| S287,290, 301D | | CCACCACCATTAAATACAGATACCCCTGATCCATCCTCTTCATTCCAAGGTAGACCACCTGATACTGGTTTCAATACTCC | | GGAGTATTGAAACCAGTATCAGGTGGTCTACCTTGGAATGAAGAGGATGGATCAGGGGTATCTGTATTTAATGGTGGTGG |
| S335,339D | | GGTGGAGCTGCAAACAATCGTTTAGATGTCCATAACGATGCTCCAATTGTTGCTGCTC | | GAGCAGCAACAATTGGAGCATCGTTATGGACATCTAAACGATTGTTTGCAGCTCCACC |
| S384, 388, 389D | | CCAAAAGCATCCGGTGCTCGTGATGATCTTCTCGATGATATTATGCAAGGTATGGC | | GCCATACCTTGCATAATATCATCGAGAAGATCATCACGAGCACCGGATGCTTTTGG |
| VCA domain  S-D | | GTATTGCTTGGGCTGGTGATGATGATGACGATGAAGATGAAGATGATGATGATGATTGGGATTAAACTAGT | | ACTAGTTTAATCCCAATCATCATCATCATCTTCATCTTCATCGTCATCATCATCACCAGCCCAAGCAATAC |
| **Oligonucleotides used in WAVE2 cloning and Site directed mutagenesis** | | | | |
| WAVE2 | GGTACCGCCACCATGCCGTTAGTAACGAGGAAC | | | TCTAGATTAATCGGACCAGTCGTCCTCATCAAATTC |
| S293/296/298/308A | GGACCCAAAAGATCCGCTGTGGTCGCCCCAGCCCATCCACCACCAGCTCCTCCTCTAGGCGCTCCACCAGGCCCTAAACC | | | GGTTTAGGGCCTGGTGGAGCGCCTAGAGGAGGAGCTGGTGGTGGATGGGCTGGGGCGACCACAGCGGATCTTTTGGGTCC |
| S343/351A, T346A | CCTGTAGGATTTGGGGCTCCAGGGgCGCCTCCACCACCCGCACCCCCATCTTTCC | | | GGAAAGATGGGGGTGCGGGTGGTGGAGGCGcCCCTGGAGCCCCAAATCCTACAGG |
| S429/S442A | ACCAAGCCCAAGTCCGCCTTGCCTGCCGTGAGCGATGCCCGTAGCGACCTGCTTGCAGCCATCCGTCAAG | | | CTTGACGGATGGCTGCAAGCAGGTCGCTACGGGCATCGCTCACGGCAGGCAAGGCGGACTTGGGCTTGGT |
| Oligonucleotides used in CRISPR/Cas9- mediated deletion of WAVE1 and WAVE2 | | | | |
| Cas9-targeting sequence WASF1: | | | GGCTGAGCTCAAGATGCCGT | |
| Cas9-targeting sequence WASF2: | | | GTGCCTTGGCTCGATGTTCC | |
