# Supplementary material for: Cell–substrate adhesion drives Scar/WAVE activation and phosphorylation by a Ste20-family kinase, which controls pseudopod lifetime
Source: PLoS Biol. 2020 Aug 3;18(8):e3000774. doi: 10.1371/journal.pbio.3000774 (PMC7425996; doi:10.1371/journal.pbio.3000774)
Supplement: S4 Table — LC-MS/MS, Liquid chromatography-tandem mass spectrometry. (DOCX) [file pbio.3000774.s021.docx]

# S4 Table: List of phosphopetides detected by LC-MS/MS

| **Enzyme** | **Start** | **Stop** | **Phospho-Peptide sequence** | **Modification** | **Mascot Ion score** |
| --- | --- | --- | --- | --- | --- |
| Trypsin | 75 | 90 | (R)IRPLIQSIPSIED**y**HR(N) | Phospho (+80) | 60.1 |
| Trypsin | 113 | 131 | (R)NQHFTHASIPASINTV**y**EK(C) | Phospho (+80) | 61.0 |
| Trypsin | 208 | 217 | (K)VR**y**DPVTGEK(I) | Phospho (+80) | 45.5 |
| ChymoTrypsin | 278 | 295 | (Y)TPPPPPLN**tst**PSPSSSF(Q) | Phospho (+80) | 34.6 |
| ChymoTrypsin | 278 | 295 | (Y)TPPPPPLNTSTP**s**PSSSF(Q) | Phospho (+80) | 37.6 |
| Trypsin | 384 | 402 | (R)SDLL**ss**I**m**QG**m**ALKPAEER(K) | Phospho (+80), 2 Oxidation (+16) | 43.3 |

Residues in red and blue are detected phosphorylated.
