# Supplementary material for: Cell–substrate adhesion drives Scar/WAVE activation and phosphorylation by a Ste20-family kinase, which controls pseudopod lifetime
Source: PLoS Biol. 2020 Aug 3;18(8):e3000774. doi: 10.1371/journal.pbio.3000774 (PMC7425996; doi:10.1371/journal.pbio.3000774)
Supplement: Attachment — Submitted filename: Singh et al. full rebuttal.pdf [file pbio.3000774.s022.pdf]

Answer to reviewers.

Reviewer #1:

This manuscript reports a study of phosphorylation site identification and function analysis for the actin protrusion regulator Scar/WAVE complex. From their data, the authors conclude that cell:substrate adhesion triggers phosphorylation of Scar/WAVE, which then serves as a degradation signal. While this concept is very attractive, the authors fall short of providing convincing evidence for such a mechanism.

Major issues:

- The manuscript is completely lacking a materials and methods section, therefore making the experiments difficult to understand and not possible to be reproduce by others. It is imperative that such sections are included, including all materials used and experimental details, to better understand and appreciate the data presented.

We're sorry – this was not included because of an oversight – we submitted another paper around the same time which required a separate "STAR methods" section, and it confused us for this paper. It's now included.

- A critical issue is the sole use of band shifting analyses to evaluate phosphorylation of Scar/WAVE. This is not a sensitive phosphorylation detection method and far from quantitative. The authors need to confirm their findings using radiolabelled phosphate incorporation into the Scar/WAVE proteins, which will provide a much more quantitative way of measuring phosphate incorporation and identifying sites. One cannot conclude anything for certain with only band shifts without confirming the accuracy of the interpretation using a different, more quantitative and accurate method, to at least validate that the bands intensity and/or size reflect the phosphorylation events that are claimed.

This objection is simply incorrect, on both counts.
"Not sensitive" would mean it was not able to detect physiological levels of phosphorylation. The assay is plainly sensitive enough; almost every figure shows substantial levels of phosphorylation.
"Far from quantitative" would mean that we couldn't obtain measured numbers, or that the measured numbers were not accurate. However with our low-Bis gels we get clean, baseline separation of phosphorylated and unphosphorylated Scar/WAVE. They are transferred together, probed together, and can be directly and quantitatively compared. Bands with different numbers of phosphates are cleanly resolved from one another. This is obviously better than radiolabelled phosphate, in which the unphosphorylated protein cannot be detected, so the proportion of phosphorylation cannot be measured.

- Fig 3, The data in Dictyostelium suggesting that ERK is not responsible for the Scar band patterns, nor that these Scar species are regulated by the chemoattractants Folate and cAMP, is compelling. However, the authors absolutely need to consider previous observations from different groups that WAVE2 is phosphorylated in an ERK-dependent manner and discuss in a constructive manner. E.g. different conditions used: previous studies starved the cells and then

assessed phosphorylation after stimulation. Here, the authors presumably used resting cells (methods information lacking!), treated with a MEK inhibitor and assessed band profiles. In addition, different cells were used. They need to reconcile all the data that is now known on this, and attempt to make sense of it. It may all be valid, just different effects in different conditions; different sites were studied and identified; different roles depending on stimulus; etc.

The existing literature states that Scar/WAVE phosphorylation is mediated by MAP kinases and is essential for its activation. Our main point is that this is not true; (a) Scar/WAVE that isn't phosphorylated is fully active; and (b) in both the Dictyostelium and mammalian cells we studies, Scar/WAVE is not phosphorylated by Erk2 and is fully active. Thus our main point is true - Erk2 phosphorylation is not required for Scar/WAVE activity.
What we do not claim, nor would ever want to claim, is that MAP kinase phosphorylation never occurs. We have now added a sentence emphasizing this.

In addition, contrary to what the authors state multiple times in their manuscript, it is highly possible that the identified phosphorylation sites be targeted by ERK1/2. The authors appear to have a misconception about the ERK1/2 consensus sites. ERK1/2's minimal consensus motif is SP/TP, and a longer consensus motif was defined as PXSP/PXTP. However, in s previous study showed that these motifs were neither necessary nor sufficient to identify ERK substrates. In fact, some of the previously identified and validated ERK targets are phosphorylated on sites that even lack an adjacent proline in the +1 position (Carlson et al Sci Signal 2011). The authors need to revise their comments, interpretations, and conclusions accordingly.

The (excellent) Carlson paper identifies 100 peptides from 80 proteins that are phosphorylated by ERK2 (we note, incidentally, that no WAVE2 phosphorylation was seen, even though WAVE2 is abundant). A single one of these proteins has ERK2 phosphorylation sites without a P in the +1 position, as well as other ERK2 sites that fit the consensus. We have therefore qualified our comments as requested, but our conclusion is supported by multiple strands of evidence. The new text makes this clearer.

- Fig. 4, With the data presented, the authors cannot conclude that cell:substrate adhesion causes activation of Scar phosphorylation, they can only conclude that they observe a correlation between the two. The authors should, in fact, also evaluate cell:substrate adhesion of each cell line.

As described in the cover letter, we have removed the mammalian cell lines because referees found the data less convincing.
As for "cause" – we have softened our language, but it is not true that we just observe "correlation". We change the adhesion, and following the change we always observe a change in Scar phosphorylation. When we make nonadherent cells adhere, the process is followed by an increase in phospho-Scar. When we suspend adherent cells, a decrease in phospho-Scar follows. When we examine mutants that cannot adhere they have little or no Scar phosphorylation, unless we force them to adhere, when phosphoScar is seen. Throughout the biological literature this would be accepted as "cause" rather than "correlate".
We cannot show all details of the pathway, so we cannot claim it "directly causes" phosphorylation, but that would be a long way beyond the scope of this work.

- Fig. 5, The authors need to better analyze the migration phenotype of their cells. The ScarS8A mutant clearly makes larger protrusions, which may reflect an important role of the phosphorylation. Similarly, the WAVE2S8A looks like a keratocyte with a very large lamellipod. The authors need to consider these phenotypes and analyze in more detail.

We agree. We have therefore substantially increased the analysis of cell movement and pseudopod dynamics. We split fig 5 from the former submission into figure 5 (showing the phosphorylation mutants are functional) and Fig. 6 (quantitating the differences in pseudopod behaviour)

- Fig. 7. The data presented are not sufficient to conclude that Scar/WAVE2 phosphorylation is a degradation signal. If phosphorylation was a degradation signal, S8A should have a longer half-life. To properly study the lifetime of proteins, the authors need to do pulse chase experiments. In addition, significance of the variations reported in 7D and 7E need to be determined.

The referees were unhappy with this figure, so we've removed it. We think the evidence suggests it's correct, but suspect that to prove it soundly would take a complete paper.

We have replaced the degradation figure with the identification of the kinase, which is much clearer.

Minor issues:

- There is too much discussion and unnecessary attempts to justify the work in the introduction section. The discussion needs to be moved to the appropriate section.
Moved as requested
- To report quantified data, SEM should not be used. SEM is not a descriptive statistic and should not be used to report data variability. SD should be used, or even better the 95% confidence interval.
We have used the wrong stats in some figures, so apologies.
We use SEM to measure our confidence that two means are significantly different and SD to describe variability in the data. We believe SD is now used where descriptive statistics are appropriate (there was one section that was described incorrectly in the text) but would be glad to know details of any further incorrect uses.
- The authors need to define Mccc1, explain why it is used as a control and perhaps add a reference.
This has been added to the methods section, referenced, and introduced in the text.
- The authors should not call the S8A mutants "unphosphorylated" since there is still phosphorylation detected in these mutants. They should either use the S13A mutants, or better define the S8A mutants.
Agreed. We now call them "phosphorylation-deficient".
- Fig 1G, The authors state that deletion of the VCA domain did not reduced the number of bands, but it clearly reduces the intensities, though. Therefore, it does seem that phosphorylation is reduced in this mutant. The authors need to explain. As they themselves wrote, "only looking at the bands can lead to an underestimation of the total change in phosphate because the higher bands may contain several phosphates".
Our key point is that we have not removed the number of binding sites that can be

phosphorylated. Of course the intensities are changed – VCA mutants are completely different biologically – but the conclusion's not altered. We've added a brief clarifying note.
- Fig 1F, the description of the phosphorylation sites previously identified and reported in this study is lacking description of Ser 335 and 339.
Um – as far as we know this is the first description of those sites?
- Fig 2 Clever use of RacGEF inhibitor, Pir121 mutant and latrunculin to investigate causal relationship between phosphorylation and localization of Scar. However, localization cannot be used in lieu of activation. The authors can propose a model of phosphorylation before activation, speculating that localization reflects activation, but they need to make it clear that it is not really known.
We think we have been very clear. The (original) text reads "It is interesting to note that there is currently no biochemical assay for Scar/WAVE activity. The only measure of activation is recruitment to the extreme leading edge, and downstream consequences such as actin polymerization. This phosphorylation assay could therefore be a potentially useful indirect measure of recent Scar/WAVE activation *in vivo*.".
We have now added to this, but it was not ambiguous.
- Fig. 4L, 4N, 4O, need to show graph that represents at least 3 independent experiments with deviation, there are no error bars.
These figures have now been removed (see above).
- Fig 5H the authors cannot conclude anything about the activation or inactivation of Scar because they have no way of assessing activity. They need to conclude about what they can measure, the data they obtain.
Don't really understand this. Cells with no Scar/WAVE make no pseudopods or lamellipods. Cells with hyperactive Scar/WAVE make larger pseudopods and lamellipods. Cells with inactive Scar/WAVE make no pseudopods or lamellipods.

Maybe the query is with the word "activated", as opposed to "active"? because the Scar/WAVE is plainly working, ie active. We've made that change to this section. If the referee meant something else could they please be more specific?
- Fig 6A. Control cells need to be included to compare the levels of Scar downregulation in the presence of doxycycline.
The first two lanes are the control cells. They were lysed after 48h of Doxycycline addition/removal. The -dox lane shows essentially no Scar.
- Page 17 line 409: … in the proline-rich domain (WAVE2S8A/T1A). This mutant also includes mutations in the V domain.
Corrected, thank you.
- The authors report ScarS13A and S13D mutants and F-actin accumulation in pseudopods of cells in supplementary video 6, but they also need to show the WT Scar to compare in this experiment.
The first cell of Video is WT Scar.
- The experiment of modulating adhesion by changing confluency is not useful, can be misleading, and should be removed. Many other factors can affect adherence to substrate, including cell:cell interactions and repulsions (contact inhibition), confluence-induced secretion of factors, etc.
We have removed this section as requested.
- By mutating multiple sites and then analyzing Scasr/WAVE function, they are likely affecting multiple processes, which are likely differentially regulated by distinct phosphorylation events. For example, the morphological and migratory phenotype of cells with Scar/WAVE2

phosphomutant is likely the result of disturbing multiple phosphorylation sites that are liklely targeted by different kinases and have different regulatory roles. The authors need to consider these possibilities.
We agree. We've added a sentence to the discussion to make this clear.
- The authors should discuss potential mechanism through which mechanism coupling could possibly lead to activation of Scar/WAVE, including which kinase could be involved.
We have done better than this – we've identified a major kinase.

Reviewer #2:

This paper from Singh et colleagues sheds light on the turnover of the Scar/Wave complex in an integrated study in Dictyostelium and mammalian cells. The Scar/WAVE complex activates the Arp2/3 complex to generate protrusions such as pseudopods or lamellipods. Like all signalling pathway, inactivating the Scar/WAVE complex is as important as activating it for physiology, yet very little was known about this until this paper.

The authors first show that Scar/WAVE is mostly phosphorylated in the poly proline region and identified the specific sites involved. Then, through a series of elegant experiments, they make the important finding that phosphorylation happens after Scar/WAVE recruitment/activation at the lamellipodia. Interestingly, this activation-mediated phosphorylation was not stimulated by MAP kinases, but very strongly stimulated by substrate adhesion. While phosphomimetic and unphosphorylatable Scar mutants can perfectly rescue Scar null cells (meaning Scar phosphorylation has nothing to do with Scar activation), these mutants have phenotypes in protrusion lifetime and cell migration. Last, the authors nicely show that Scar phosphorylation increases its degradation rate, in good agreement with the increased turnover of protrusions. Altogether, this story changes our view of the role of Wave complex phosphorylation and proposes a new paradigm where substrate adhesion promotes Scar/WAVE turnover.

I think this is an excellent paper that meets the very high standards of PloS Biology. The paper is very well written, the data are very convincing and accurately quantified when appropriate. I highly recommend publication providing that the authors add an appropriate method section and answer to my minor comments.

Thank you. We hope this is suitable.

Comments:

1) Western Blot quantification
While I appreciate the care of the authors to quantify their western blots, the method they used is not consistent and sometime confusing (probably the reason for this was explained in the missing methods section). I think homogenising this would help the reader. Few instances:

i) in Figure 2C the results are quantified as a percentage of phosphorylation (« ratio of upper (more phosphorylated) band aggregate intensity to total Scar »). But then in Fig 2H, the authors switch to an intensity ratio « upper/lowest ». Why is it not possible to also do a percentage: upper/(upper+lower)*100 ? I understand the concern of the authors that this « underestimates the total change in phosphate because the higher bands that are disproportionately lost contain several phosphates”. But in this example, I don’t think this applies as in mammals, Scar essentially has only two bands (except in in Fig 4 KL).

We hope the methods section makes it clearer. We don’t think any of the Dicty or mammalian westerns are simple enough to reduce to two bands; the data always seem to show several. We hope that now we removed the adhesion section, which showed the most mutually incompatible results, this is clearer.

ii) Similarly, in figure 4C, there are labels « a » and « b » but they are not referred to in the legend (I supposed the authors did the ratio between the integrated intensity of the signal in a and the sum of the integrated intensity of the signal in a plus b, but no way to be sure).

Sorted, thanks

iii) Last, the relation between the gel and the quantification in Fig 4 KL is confusing, because it's not clear which bands the authors considered for their ratio (the legend refers to an arrow, which is missing on the figure). I think it's rather clear that JVM3 and Jurkat have more of the lower band than Cos7 cells, but B16F1 sample also has two bands. Does the lower band in the B16F1 sample corresponds to the upper band in the JVM3 sample? Also, there are vertical lines between bands on the WAVE2 panel, which are weird. Again, a simple percentage of the amount of Wave present in the various state may help (maybe would need 3 states instead of two in this panel to account for the low phosphorylation state of suspension cells)

These are the blots we have removed. WAVE2 is completely different in the adherent and nonadherent cells. If they confuse this referee (and they certainly confuse us) they are not ready for typical readers.

2) Expression levels of Scar mutants in Scar null cells

Most of the Dictyostelium work is based on rescuing Scar null cells with WT or mutant Scar, which is obviously the gold standard here, as, as the authors correctly point out, the key to Scar regulation is the Scar complex (Fig 6A). However, the authors state (line 425) "As usual (46) both mutants express at wild type levels (Figure 6A)". This formulation is confusing as it may either mean that the WT, SD and SA Scar versions express at a similar level, or that the levels of expression of the WT Scar (or SD/SA mutants) in Scar null cells are similar to the level of endogenous Scar in Ax3 cells. While it is clear in figure 6A that WT, SD and SA Scar versions express at a similar level, it seems that this level is higher than endogenous Scar (compare lane 1 to lane 3 in the Scar blot in Fig 6A). In addition, unless I'm mistaken, the Blagg et al. Current Biol 2003 paper does not compare the levels of Scar between WT cells and Scar null cells rescued with Scar. So, I would clarify this statement.

In Dictyostelium (less so in mammalian cells) the quantity of Scar protein is remarkably robust to over-expression. Figure 6A is a bit of an outlier – we never normally see that high a proportion of Scar in the upper bands. We have added supplementary figure 4 to address the point directly.

a) I actually think it would be important to know if these rescued cells show any upregulation of the other members of the complex (that is, do you have « free » Scar in them), and if the percentage of phosphorylation of endogenous Scar in Ax3 cells is identical to WT Scar in the rescued Scar null cells (the band pattern in Fig6A is similar, but do you have more unphosphorylated Scar in the rescued cells ?). Indeed, if the model is right, then only Scar that has been activated can be phosphorylated. But Scar localization at the lamellipodia requires Rac, ergo Pir121 (fig 2) so you expect that if there is some Scar that is not associated with Pir121, it won't go to the lamellipodia and thus won't be phosphorylated and thus won't be degraded. And thus, there is a possibility that in Figure7, the authors are underestimating the destabilising effect of their S8D mutant because the "free" Scar. A simple Pir121 blot of the gel in Fig6A and/or Fig 7C would address the question.

Obviously, this does not invalidate any of the findings. I just think it might help make the effect in Fig.7 more striking.

It's a very interesting point. We have never been able to find any free Scar in Dictyostelium, by a range of methods; they must have a superstrength mechanism for getting rid of it. I would love to get a grant to study this, but we live in very conservative times. Again, we can find free

Scar/WAVE when we overexpress it in mammalian cells. Anyhow, to answer the referee's question – we tested this in detail using immunoprecipitations to make supplementary figure 4. The small amount in the supernatants is presumably due to incomplete IP or complex denaturation during the prep; in any case it is no different between WT and Scar overexpressers. We have removed the figure 7 data.

3) Other minor comments/suggestions/typos

-The last figure shows that the Scar S8D mutant show a higher protein turnover rate than the WT, which fits with in vivo observation. I think this is a very important result, and the quantification is convincing. However, the panel the authors chose to display this is not very convincing (it's not obvious that there is a difference between the S8A and the S8D mutant for instance). I would suggest the authors to use another panel from their quantification.
We removed this part of the paper in response to other referees, but will remember when we submit the stability in the future.
-Linescans in figures (like Fig1A for instance) have no unit, it's just written « distance ». It's obviously not pixels (would be fine if it were in pixels, it's just weird not to have a unit).
Pixels would be possible but would focus on the scanning equipment. Distance in mm is different from blot to blot, so not appropriate. We've therefore changed it to AU – hope this sorts the weirdness enough.
-Page7, for clarity, it might be worth moving the statement about the fact that the sites in human WAVE2 do not fit the MAPK consensus to Page9 when the authors confirm their Dicty findings to human WAVE2.
Have done. Is this clearer now?
-Page9, line 208 « to almost nothing ». Without quantification, I don't think it's fair to say this. I don't think a quantification is needed for this as this is just a control that a known inhibitor is working. I would just remove this.
Removed
Figure 7A and B
Maybe the authors could remind the reader of the phosphorylation state of Scar/WAVE in the conditions depicted in this panel…
We have removed these panels
-Table1 I would change « By MS and Screening" into "this study (MS and Screening)" for the Scar residues.
Good point. We have cleaned up that table in general, as well.
-Line 405 : the sentence should read « Scar S13A and Scar S13D »
-Line 409 : The « S » in WAVE2 S8A/T1A has to be superscript
Both corrected.

Reviewer #3:

Singh and colleagues studied the role of serine phosphorylation of SCAR/WAVE, a conserved Arp2/3 activator driving cellular protrusions in eukaryotic cells. Here, the authors followed up on their previous studies on the basally phosphorylated C-terminal VCA domain required for pseudopod dynamics in Dictyostelium. Using similar biochemical approaches and rescue experiments with phosphomutant and phosphomimetic SCAR/WAVE variants they found that serine phosphorylation of the proline-rich region of the SCAR/WAVE protein is largely dispensable for both complex stability and activation of the SCAR/WAVE complex. Phosphomutant and phosphomimetic SCAR/WAVE variants are fully functional, while the phosphomimetic Scar is less stable. Finally, the authors found that phosphorylation of SCAR/WAVE seems to be independent from chemotactic cues such as cAMP but rather promoted by cell-substrate adhesion.

Overall, the manuscript is clearly written; most experiments are carefully conducted and well documented. It also addresses a very exciting question in the actin and cell migration field as well. There are indeed numerous in vitro data on SCAR/WAVE phosphorylation in both vertebrates and invertebrates, but the physiological relevance in vivo is still unclear. Unfortunately, the novelty of the findings in this work is still very limited, and the title of this manuscript promises much more than most of the negative results could exclude. This is also true for possible serine kinases that might phosphorylate SCAR/WAVE in vivo. Erks as well-known candidate kinases could be excluded, but the question which kinases really contribute to the observed cell-substrate-induced phosphorylation of endogenous SCAR/WAVE still remains unclear. It is also unclear, if and how cell-cell adhesion could affect SCAR/WAVE phosphorylation in a physiological context. Thus, the author's final statement in the discussion "We look forward to seeing new biological examples emerge" hits exactly the point, the manuscript in its present form lacks exceptional originality and relevance for publication in PLOS Biology.

We have now identified a kinase. The rejection of Erk2, and the exclusion of phosphorylation in general as an activating process, are absolutely fundamentally important to the actin polymerization field. The adhesion data are clear; I'm not sure what "physiological context" the referee is asking for, please clarify.

1) The authors found a simple but very efficient way to improve separation of different phosphoforms of the endogenous SCAR/WAVE protein using low bis-acrylamide gels. This dramatic improvement of SDS-PAGE is not always so well visible, e.g. fig. 3D upon cAMP induction or fig. 4K, 3K, 3M.
Starved Dictyostelium cells migrate very rapidly and seem to turn over Scar much faster; they also stick less to the substrate. Thus it's probably not "less visible" as much as "less phosphorylated overall".
2) Figure legend 1L: „Unlike ScarWT, ScarS8D shows little removal of additional bands after phosphatase treatment". This is not visible, or do the authors mean Scar8A?
Clarified
3) Does loss of rac1 also result in a strong reduction of SCAR/WAVE phosphorylation?
Loss of Rac1A is nearly lethal (the mutant was described at a meeting but is not available); in any case there are numerous other isoforms. The combination of inhibitors and targetted

mutations of PIR121's Rac binding sight are unambiguous, however.
4) Pak-CRIB-mRFP-mars intensity should be quantified (fig. 2I).
Done
5) Hyperphosphorylation observed after Latrunculin A treatment should be analyzed in more detail in the first 10 minutes fig. 2L since the recruitment of the SCAR/WAVE complex is already obvious after 60 seconds (fig. 2K). Please also show the localization of different leading edge proteins such as Ena/Vasp or Abl as controls (fig. 2K).
New assays have been performed with the requested time course (0, 2, 5,10, 20, 30 mins).
Arp2/3 complex has been included as a control
6) Quantification of Western blots shown in fig. 3A/B and 3D/E is unclear. How do the authors normalize? There are different phosphorylation levels (e.g. 35% and < 20%) after cAMP and folate stimulation, why is it so?
The developed cells that are competent to respond to cAMP have reduced Scar phosphorylation. Suppl. Figure 3 has been added to show this.
7) In fig. 3I and 4G, Western blots with mutant cell lysates should be probed for anti-Erks and ant-Talin.
Talin has been incorporated in the figure panel.
8) Analysis of SCAR/WAVE phosphorylation in integrin mutant cells must be included to strengthen the author's conclusions.
Dictyostelium integrin genes make up a fairly large family (SibA-E). We did not observe either reduction in adhesion or of Scar phosphorylation in sibA- cells. Rescue of Scar phosphorylation by forcing mutants to adhere is a stronger demonstration.
9) The authors hypothesize that "SCAR/WAVE phosphorylation is not required for its activation, but rather modulates cell migration by controlling the dynamics of SCAR/WAVE patches." Here, it remains completely unclear what are these patches, and what is their biological relevance in cell migration?
We have clarified this point in the text. Scar/WAVE is always organised in short-lived patches when active in moving cells. The long-lived lamellar localisation seen in B16 cells is very much an outlier.
10) Please also quantify lamellipodia formation in rescued cells (fig. 5H).
Quantified and included as additional figure 6.
11) Figure 6A: The expression of the endogenous SCAR/WAVE is completely suppressed in the absence of doxycycline, rather than in the presence; this makes no sense!?
The inducible Scar-/WASP- KO is made by knocking out WASP and expressing Scar under a tet promoter (Davidson et al., 2018). Thus you lose Scar when you remove doxycycline. We didn't initially understand why this wasn't clear (it is explained in detail) but have perhaps traced the problem to the word "inducible double knockout" so removed the term and explaineddifferently. Is this clear now?
12) Figure 6C, D: Please show the co-localization.
We're afraid this is too difficult for an experiment that is already insanely complex. Scar is not fixable so we can't use phalloidin and visualise Scar at the same time. The cells are already knocked out for two separate genes, have two different unlabelled genes expressed in them (the Tet inducer and the mutated Scar) so we can't express two more transgenes.

Reviewer #4:

Singh et al

In this manuscript, the authors revisit the phosphorylation of SCAR/WAVE proteins in mouse cells and Dictyostelium. Using publicly available mass spec data and site directed mutagenesis, they find that multiple Ser/Thr residues are phosphorylated in the poly-proline region of the protein. They find that mutants lacking these sites are capable of rescuing SCAR/WAVE function, but have a slightly longer residence time in the protrusions. Finally, they go on to show that phosphorylation might be related to protein turnover, although the data supporting this claim is not particularly strong. While the basic idea that the Rac GTPase activates SCAR/WAVE is clear, this paper points to this regulation being more complex and subtle, presumably allowing cells to tune protrusion activity. While I appreciate the effort it takes to understand the complexity and subtly of any cellular process, this manuscript falls short of providing much in the way of new or compelling information about protrusion dynamics, SCAR/WAVE regulation or any of the higher order processes that depend on these events. Thus, my enthusiasm for this work is somewhat low. Some specific feedback is outlined below:

1) The authors do not identify the kinase or kinases that are responsible for this phosphorylation. Nor do they identify the phosphatase that dephosphorylates these sites (potentially more interesting based on their model).
We have now identified and presented the kinase in the resubmitted manuscript.
We do not believe that these residues are dephosphorylated; we thing the phosphoforms are degraded.
They provide reasonable data that Erk is not responsible, contradicting a previous paper in the literature. This is negative data that could be because the previous paper was wrong or that they are just using different cell lines. Regardless of the explanation, the lack of positive information about the identity of the responsible kinase is disappointing and doesn't allow the reader to put this putative new regulation into any kind of cellular context.
As earlier – we have now shown the sepA kinase interacts with Scar, and that Scar phosphorylation falls greatly when it is lost. This makes up the new Figure 8.

Previous manuscripts concluded that Erk2 phosphorylation of Scar/WAVE was an essential part of the activation process. We show conclusively that this is not true. Scar and WAVE with all the named sites mutated are activated more than native Scar/WAVE. This would still be true if some other cells exist in which Erk2 phosphorylates the non-consensus MAPK sites, to give the same pattern of bands that we have identified as not requiring Erk2 (which we doubt, but is formally possible).

2) The authors make a big deal about how adhesion regulates this phosphorylation and, based on one knockout in Dictyostelium (talin), claim that it is not integrin-based. As far as I know, no integrins have ever been identified in Dicty, so its a bit hard to know what the talin mutant data really means.
Dictyostelium integrins are named SibA-E (similar to integrin Beta); Sibs mediate adhesion, interact with talin, and have the canonical talin interaction domains in their transmembrane helices (GxxxG) and cytosolic (2x NPLY) domains. They're called "Similar to integrin" rather

than “integrin” because the extracellular domains are different (Dicty matrix is different from collagen). But they plainly work in a comparable way.
In fact, many papers from a number of groups has shown that integrin-based adhesion regulates SCAR/WAVE through Rac and other mechanisms. Unfortunately, none of these papers are mentioned.
The integrin field is gigantic, and we’re sorry if we missed papers this referee wants cited; please suggest any examples.
One thing that was particularly puzzling to me was that SCAR/WAVE clearly regulates protrusion events on the dorsal surface of cells, far away from site of substrate adhesion. Are these protrusions not subject to this phospho-reguation?
An interesting point, and almost impossible to measure, as we can’t separate Scar/WAVE that has made lamellipods from “dorsal ruffles”. However, we have clearly show phosphoregulation is not essential for Scar/WAVE to function in pseudopods; we don’t think it fundamentally affects our conclusions if we can’t study other roles of Scar/WAVE.
Some of the other experiments such as looking across a panel of adherent and suspension cell lines or letting cells get over-confluent as proof of their hypothesis about adhesion are fairly weak pieces of evidence.
In response to this referee and others, we removed the mammalian cell line adhesion panels, and replaced them with additional data on Scar/WAVE regulation and an additional figure on the kinase.
3) Another area that was notably overlooked in the paper was how phosphorylation may affect the interaction of SCAR/WAVE with other members of the WRC or Rac? This is puzzling since these authors have done some very nice work in this area in recent years.
Rac is absolutely essential for Scar/WAVE function (confirmed in this paper but shown in multiple other papers by genetics, molecular biology and with inhibitors). We show that phosphorylation is not required at all for Scar/WAVE to function, and phosphorylated Scar/WAVE also works well. We’ve therefore concluded that phosphorylation is not necessary for Rac interactions, nor stops them.
There is a potentially interesting study if it modulated Rac interactions, but it would be complicated (the Rac interacts with a different complex subunit, well away from the Scar/WAVE) and take a great deal of work to understand – the only quantitative measurements of Rac affinity for Scar/WAVE have used bacterially reconstituted, truncated mini-WAVE.
Also, I was a bit surprised that the authors elected to use the supposed Rac inhibitor, EHT1864. This drug has a terrible reputation in the field and likely has a number of off-target effects. At the very least, they should have used one of the many other approaches that are available to address this point.
The data that uses the inhibitors is internally supported strongest way possible, by using targeted mutations in the Rac-binding domain of PIR121. The mutations and the inhibitor agree perfectly, meaning it’s extremely unlikely to be due to an off-target effect.
Additionally - this is unfair refereeing. There have been more than 100 papers using this paper since 2019, including one of ours that measured the effect on Rac (so not a “supposed” inhibitor) and reversibility. If a referee wants to say that a drug has off-target effects or a terrible reputation they need to support it with data or publications, not hearsay.

4) The effects on protein turnover presented in Figure 7 are not very impressive. Nor is the mechanism of this turnover addressed, although they state that they don't think ubiquitinylation is involved. The time scale of the degradation also doesn't seem to fit with the cellular effects (minutes/hours vs seconds).

Our data have consistently pointed at this mechanism for some years, but as is clear from the lack of ubiquitin in mass spec and banding patterns (we have done, but not shown, experiments with full DUB protection), it is clearly not ubiquitin. It's also fully consistent with the biological data (the unphosphorylatable Scar/WAVE is more active and lasts longer).
But we agree with the referee, these results are not clear-cut enough. A strong proof is currently far beyond the scope of this paper, so we have removed the figure and replaced the data with the kinase work.

5) The functional effects of this phosphorylation event are not very well explored. There seems to be a minor effect on lifetime of SCAR/WAVE at protrusions, but how this affects the protrusion themselves is not really described. Moreover, if a cell has a different lifetime of SCAR/WAVE at protrusions, what is the effect on overall cell motility? Again, these questions are largely unanswered by this work.
These issues have been explored and added as figure 6 and additions to figure 5.
