# Supplementary material for: Cell–substrate adhesion drives Scar/WAVE activation and phosphorylation by a Ste20-family kinase, which controls pseudopod lifetime
Source: PLoS Biol. 2020 Aug 3;18(8):e3000774. doi: 10.1371/journal.pbio.3000774 (PMC7425996; doi:10.1371/journal.pbio.3000774)
Supplement: Attachment — Submitted filename: Rebuttal2.docx [file pbio.3000774.s024.docx]

Rev. 1:
The authors have appropriately responded and addressed the reviewers' comments and concerns.


Rev. 3:
My major concern about the first manuscript version was the quite limited novelty of the findings, particularly the missing functionally relevant serine kinase that phosphorylates SCAR/WAVE in the described cell adhesion-dependent context. In the revised version the Insall group now provided strong evidence that the Ste20 group kinase, SepA contributes substantially to SCAR/WAVE phosphorylation, an exciting novel finding that strengthens the whole story.

Thus overall, I am satisfied with the improved version of the manuscript. I only have two remaining minor points. First, the authors should include the SepA kinase into manuscript title.

We have added the kinase to the manuscript title. We used a generic name to maintain the generality of the work, which describes human as well as Dictyostelium cells.

Secondly, the authors should further discuss their findings and possible conserved functions of Ste20 like kinases in SCAR/WAVE regulation in more detail.

We have added a more detailed discussion of the MAP3Ks. They are legion, very little is known about them, and none stand out as the “best” homologues, so we have not discussed pathways leading to them (as we would have if they were PAKs, MLKs or Rafs for example). We also removed an earlier statement that we did not know the kinase; it had been kept in error.


Rev. 4:
In the revised version of this manuscript, the authors have somewhat improved the presentation of their story by eliminating some of the more poorly conceived experiments and adding some new data such as the identification of an orphan kinase that phosphorylates Scar in Dicty cells. In the end however, this story is just not that interesting. They accomplish two things: 1. showing the Erk is not a major kinase for Scar/Wave and 2. Phosphorylation has a minor effect on pseudopod stability that does not apparently have much affect on whole cell motility or the fidelity of directed migration. While aficionados of Scar/Wave biology may be interested in this, I doubt that this will really have much influence on the field of actin biology or motility.
